# Milestones in Heart Failure: How Far We Have Come and How Far We Have Left to Go

**DOI:** 10.7759/cureus.20359

**Published:** 2021-12-12

**Authors:** Harini Gajjela, Iljena Kela, Chandra L Kakarala, Mohammad Hassan, Rishab Belavadi, Sri Vallabh Reddy Gudigopuram, Ciri C Raguthu, Srimy Modi, Ibrahim Sange

**Affiliations:** 1 Research, Our Lady of Fatima University College of Medicine, Valenzuela, PHL; 2 Family Medicine, Jagiellonian University Medical College, Krakow, POL; 3 Internal Medicine, Jawaharlal Institute of Post-Graduate Medical Education and Research (JIPMER), Pondicherry, IND; 4 Internal Medicine, Mohiuddin Islamic Medical College, Mirpur, PAK; 5 Surgery, Jawaharlal Institute of Post-Graduate Medical Education and Research (JIPMER), Pondicherry, IND; 6 Research, Tianjin Medical University, Tianjin, CHN; 7 Research, K. J. Somaiya Medical College, Mumbai, IND; 8 Research, California Institute of Behavioral Neurosciences & Psychology, Fairfield, USA

**Keywords:** heart failure with preserved ejection fraction (hfpef), hfref, review of clinical trials, angiotensin neprilysin inhibitor, ace inhibitors and angiotensin receptor blockers, beta adrenergic blockers, heart failure

## Abstract

Heart failure is a clinically complex syndrome that results due to the failure of the ventricles to function as pump and oxygenate end organs. The repercussions of inadequate perfusion are seen in the form of sympathetic overactivation and third spacing, leading to clinical signs of increased blood pressure, dyspnea, fatigue, palpitations, etc. This article provided a brief overview of the clinical syndrome of heart failure; its epidemiology, risk factors, symptoms, and staging; and the mechanisms involved in disease progression. This article also described several landmark trials in heart failure that tested the efficacy of first-line drugs such as beta-blockers, angiotensin receptor blockers, angiotensin-converting enzyme inhibitors, and the latest drugs in the field of heart failure: angiotensin receptor neprilysin inhibitors. Most studies described in this article were guideline-setting trials that revolutionized the practice of medicine and cardiology.

## Introduction and background

Heart failure (HF) is a complex, heterogeneous clinical syndrome that develops due to structural, functional, or physiologic abnormality of the heart, leading to a gradual and progressive failure of the ventricles to fill and to pump blood [1]. Although descriptions of HF were made in ancient literature, the invention of the electrocardiogram (EKG) made a significant impact in recognizing and identifying the condition [2]. Today, HF is a fairly prevalent diagnosis, as it affects more than six million Americans over the age of 20 [3]. This prevalence is projected to rise to 46% by 2030, affecting eight million Americans [4]. Although the prevalence is higher among men than women, the HF mortality rate is significantly higher for women [2]. Many clinicians use the ejection fraction (EF) to distinguish between the systolic and diastolic types of the condition, i.e., HF with reduced EF (HFrEF), the systolic type, and HF with preserved EF (HFpEF), the diastolic type [1]. The crucial highlights and differences between these conditions are presented below in Table 1.

**Table 1:** Systolic vs. diastolic heart failure EDV: end-diastolic volume; HF: heart failure; SV: stroke volume

Parameter	Heart failure with reduced ejection fraction (HFrEF)	Heart failure with preserved ejection fraction (HFpEF)
Ejection fraction (amount of the blood pumped i.e. SV, per a single ventricular contraction at the end of diastole i.e. EDV. Expressed as a percentage by multiplying the ratio by 100)	≤40 %	≥50%
Also known as	Systolic HF	Diastolic HF
Ventricular changes	Primarily involves dilation of the left ventricular chamber resulting in weak contraction	Ventricular hypertrophy causes “stiffening” of the walls and impairs diastolic filling

More recently, patients with an EF ranging from >40% and <50% have been placed into the HF with the mid-range EF (HFmEF) group [5]. The presence of hypertension, diabetes mellitus (DM), metabolic syndrome, tobacco use, and chronic alcohol use are all established risk factors that accelerate the development of HF [5]. There are a wide variety of causes for structural and functional heart diseases, which ultimately result in the clinical syndrome of HF. Structural causes such as dilated cardiomyopathy, familial cardiomyopathy, and inflammatory causes, such as myocarditis, make up a significant number of HF cases [1]. It is noteworthy to emphasize that despite recent advances in medicine, HF remains a clinical diagnosis. Therefore, a well-accounted history and physical examination remain the cornerstone in diagnosing HF [1]. The goal of history taking must be aimed at narrowing down and determining the cause of HF. The physical examination must be sought to assess cardiac function and volume status and to determine the severity of symptoms [1]. The history and physical examination findings can further be aided by the New York Heart Association (NYHA) and the American Heart Association (AHA) criteria [1,6]. These classification systems take into account EF, the signs and symptoms of the patient, and their quality of life [1,6]. On initial evaluation, patients suspected of HF must undergo comprehensive screening, including complete blood count (CBC), lipid profile, serum glucose, assessment of renal and hepatic function, including urinalysis (UA), serum electrolyte levels, serum creatinine, metabolic panel, electrocardiogram (EKG), and echocardiography [1,7]. Management of HF highly depends on the cause and severity of symptoms. It involves non-pharmacologic and pharmacologic interventions, such as but not limited to diet, reduced salt, and water consumption, exercise, angiotensin-converting enzyme inhibitors (ACEIs), angiotensin receptor blockers (ARBs), beta-blockers (BBs), angiotensin receptor neprilysin inhibitor (ARNI), and thiazides [1,8]. Clinicians often find themselves adjusting and sifting through different medication classes to treat HF since the pharmacotherapy varies with patient profile, the severity of HF, and adverse reactions. The purpose of this article is to: 1. Explore neurological, cardiac, and end-organ remodeling occurring due to HF; 2. Discuss the various pharmacotherapies available for HF with reduced EF with a predominant emphasis on three specific classes of drugs - BBs, ACEI/ARBs, and neprilysin inhibitors, with respect to patient factors, mortality benefits, and side effects.

## Review

Dissecting the neural and hormonal components involved in HF

When an organ undergoes stress on an acute or chronic timeline, it creates an adaptative response to maximize its function. For cardiac tissue, one of the immediate adaptative responses is the activation of the adrenergic/sympathetic nervous system (SNS), stimulating the beta-adrenergic response [9-10]. Initially, this response enhances vasoconstriction and venous tone, ultimately boosting the blood pressure and stroke volume [10]. However, such an adaptative response over a prolonged time can lead to adverse outcomes [10]. Additionally, this overactivation also causes dysfunction of chemoreceptors and baroreceptors, diminishes the parasympathetic outflow to the heart, and enhances systematic sympathetic response via epinephrine and norepinephrine spillage [11]. Together the hyperadrenergic response and overactive chemo and baroreceptor response constitute the neural component of this model.

The hormonal component of the model refers to the activation of the renin-angiotensin-aldosterone system, better known as RAAS [12]. In HFrEF, to correct the ejection fraction, the SNS activation subsequently leads to the vasoconstriction of blood vessels, including the renal arterioles, particularly the juxtaglomerular apparatus (JGA) [12]. Initially, beta-1 receptors present on the JGA enhance renin secretion upon exposure to catecholamines [12-13]. Renin, in turn, is converted to angiotensin I through angiotensinogen; furthermore, angiotensin I is converted into its potent form, angiotensin II [13]. Angiotensin II acts systematically and further works in symphony to promote and potentiate SNS effects [12]. Such effects include changes in arterial blood pressure, increased tone of the vascular system, proliferation of smooth muscle cells, inflammation, and fibrosis [14]. Intricate changes are also seen in the serum sodium levels and the extracellular fluid volume composition via the activation of aldosterone, a mineralocorticoid hormone that promotes sodium and water retention, thereby resulting in hypertension, a primary risk factor for potentiating HF [5,14]. Notably, this physio-pathological pathway has been studied to a great level, including at the molecular level of changes brought about through RAAS on sodium-potassium channels, cardiac calcium regulation, and the influence of RAAS on gene expression, etc. [14-15]. This interplay between the kidneys, heart, vascular, endocrine, and nervous systems is the driving force of the clinical syndrome of HF. At present, many therapies target blocking neuro-hormonal pathways at various points, which has become the mainstay in tackling HF.

Cardiac remodeling mechanisms

A significant change that a failing heart can make is cardiac myocyte hypertrophy, leading to ventricular enlargement [16]. This process that ultimately changes the structure, size, and shape of the cardiac myocardium is called cardiac remodeling. Numerous mechanisms have been implied in cardiac remodeling pathways, ranging from molecular to genetic to cellular [16]. The distress signals involving several intracellular molecular signaling pathways are mediated by but not limited to angiotensin II (triggering the Gq coupled receptor response), nitric oxide, direct catecholamine response (G protein-coupled responses), growth factors, etc. [16]. Mitochondrial dysfunction also accounts for the mal-adaptation seen in HF [15]. This impacts the production of adenosine triphosphate (ATP), which is the primary driver of cardiomyocyte contraction [15]. Studies have also shown that microRNAs, particularly the miR-133, have a role to play in cardiac myocyte hypertrophy [17]. Another microRNA, the MiR-208, was shown to regulate cardiac remodeling and beta myosin heavy chain expression [18]. There is ongoing intensive research on exploring these pathways in-depth to develop potential drugs to stop the deleterious effects of remodeling.

Beta-blockers and heart failure

**Figure 1:**
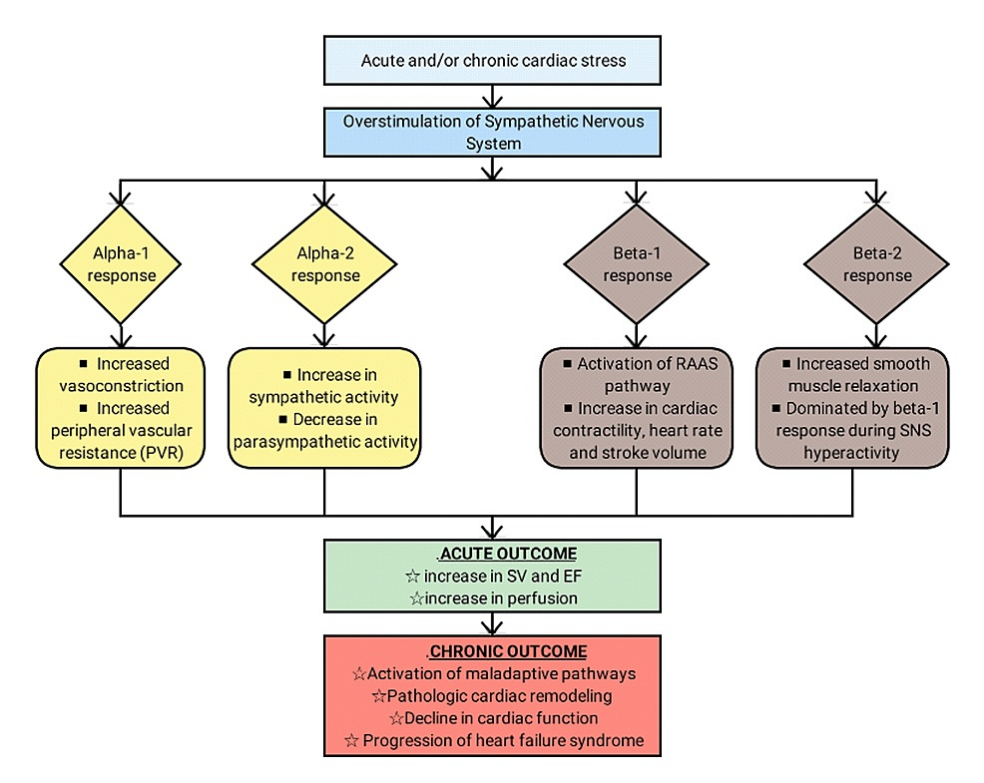
Overview of sympathetic stimulation in response to cardiac stress, leading to heart failure SNS: sympathetic nervous system; RAAS: renin-aldosterone-angiotensin-system; SV: stroke volume; EF: ejection fraction; PVR: peripheral vascular resistance

Since their invention in the late twentieth century, beta-blockers have revolutionized the practice of medicine and cardiology [19]. The SNS exerts its effects through adrenergic alpha and beta receptors [20]. In turn, these effects are mediated by the G protein-coupled receptors (GPCRs), which produce either stimulatory or inhibitory effects [21-22]. Together, the alpha and beta responses drive the sympathetic response, which is described in Figure 1.

Today, BBs are one of the principal drugs used in HF, combined with other first-line drugs [1,15]. Several landmark trials time and again have proved that BBs reduce mortality, reduce hospitalizations, and improve quality of life in HF patients, particularly in those with reduced EF.

In 1999, the Cardiac Insufficiency Bisoprolol Study II (CIBIS-II) trial, a multicenter, double-blind, randomized, placebo-controlled trial in Europe that enrolled 2,647 patients, demonstrated the advantages of bisoprolol [23]. The study enrolled patients with EF of less than 35% and belonged to New York Heart Association (NYHA) classes III to IV. A total of 1327 subjects were placed on bisoprolol 1.25 mg (milligrams), and 1320 subjects were placed on placebo. The drug was given progressively to a maximum limit of 10 mg a day. The mean follow-up accounted for 1.3 years, and the study came to a halt earlier than expected because of significant beneficial findings. The study was analyzed with an intention to treat. The primary outcome of the study showed that there was a significant decline in all-cause mortality in subjects taking bisoprolol compared to placebo (11.8% vs. 17.3%) with a hazard ratio (HR) of 0.66 (95% CI 0.54-0.81, p<0.0001). There was also a reduction in sudden deaths among patients on bisoprolol compared to placebo (3.6% vs. 6.3%) with an HR of 0.56 (0.39-0.80, p=0.0011). The study concluded that BB therapy proved beneficial among patients with stable HF. However, the study did not determine the effects of BBs on unstable HF patients.

In 2000, Berthe et al. conducted the Metoprolol CR/XL Randomized Intervention Trial in Congestive Heart Failure (MERIT-HF) trial examining the effects of metoprolol [24]. The study was designed and conducted in a randomized, double-blind, controlled fashion across 313 United States and European sites. The trial enrolled 3991 patients aged 40 to 80 years old, whose NYHA class ranged from class II to IV, who received stabilizing therapy with diuretics and ACEIs, prior to the trial. All the subjects had an EF of 40% or lower. 1990 subjects were assigned metoprolol CR/XL, and 2001 subjects were assigned a placebo. Metoprolol was initiated at a dosage of 12.5 mg a day or 25 mg a day. After the first two weeks, the dose was increased to 50 mg a day for two weeks, 100 mg a day for the next two weeks, and a dose of 200 mg a day, which is the target dose, for the final period of the trial. The subjects were followed for a mean of one year with an intention to treat analysis. The study's primary outcome measured all-cause mortality, which was found to be 0.072 vs. 0.11 per patient-year (risk reduction 0.34; 95% CI 0.19-0.47; p=0.00009), metoprolol subjects compared to placebo subjects respectively. Secondary outcomes that were reported include hospitalization due to HF or all-cause mortality and were determined as 0.16 vs. 0.22 with a risk reduction of 0.31%; 95% CI 0.20-0.40, p<0.001, cardiac-related death or non-fatal myocardial infarction outcomes were determined as 0.069 vs. 0.11, risk reduction 0.39, 95% CI 0.25-0.51, p<0.001. The study determined that in symptomatic HF patients, the use of metoprolol reduced the mortality rate.

This study was pivotal in establishing BBs as one of the primary classes of drugs used in HF. At this time, it was evident that in patients with well-controlled HF, BBs demonstrated excellent efficacy in reducing mortality rates and hospitalizations secondary to HF. However, it was not clear if BBs had the same beneficial effects in patients with HF who experience severe HF symptoms and have a significantly low quality of life. The Carvedilol Prospective Randomized Cumulative Survival (COPERNICUS) study was designed to answer this question [25]. Packer et al. conducted the COPERNICUS trial to test the benefit of carvedilol on patients with severe HF. The trial was conducted in a randomized, double-blind, parallel-group, placebo-controlled study that enrolled 2289 subjects. The subjects were randomly assigned into the carvedilol group or placebo group in a 1:1 ratio. Carvedilol was initiated at 3.125 mg or placebo twice daily, increased at two-week intervals to 6.25 mg, 12.5 mg, and then to a final target dose of 25 mg carvedilol or placebo twice daily. The mean follow-up was 10.4 months, with outpatient screening scheduled once every two months. The primary outcome of the study determined the risk of death or hospitalization. The Carvedilol group reported a 12.8% reduction in annual mortality rate compared to a 19.7% reduction with placebo. This accounts for a 35% decrease in the risk of death (p=0.00013). Carvedilol also reduced all-cause mortality or all-cause hospitalization by 24% (p=0.00004). The study concluded showing that carvedilol is effective and reduces mortality and morbidity in patients with severe HF.

Today, BBs are almost universally used in HFrEF, in combination with other first-line HF drugs [1]. Although BBs demonstrate excellent efficiency in patients with a reduced EF, research shows they may not be as effective in patients with a preserved EF [1]. Much research is underway in determining ideal drugs for HFpEF. The principal side effects of BBs include but are not limited to fatigue, bradycardia, retention of fluid, hypotension, and heart block [1]. Table 2 summarizes the studies discussed above.

**Table 2:** Trials that investigated the efficacy of beta-blockers in subjects with heart failure CIBIS II: "Cardiac Insufficiency Bisoprolol Study II"; MERIT-HF: Metoprolol CR/XL Randomized Intervention Trial in Congestive Heart Failure; COPERNICUS: "Effects of carvedilol on the morbidity of patients with Severe and Chronic Heart Failure"; EF: Ejection fraction; NYHA: "New York Heart Association"; HF: Heart failure; ACEI: Angiotensin-converting enzyme inhibitor; mg: milligram; CR: Controlled release; XL: Extended-release; BB: Beta-blocker

Name of the Study	CIBIS-II,1999 [23]	MERIT-HF, 1999 [24]	COPERNICUS, 2002 [25]
Type of study	Double-blind, randomized, placebo-controlled trial	Randomized, double-blind controlled trial	Randomized, double-blind, parallel-group, placebo-controlled trial
Number of subjects	2647	3991	2289
Mean Follow-up duration	1.3 years	1 year	10.4 months
Patient profile	EF<35% NYHA class III, IV with symptoms of HF on standard therapy with ACEIs and diuretics.	EF<40%, 40-80 years, NYHA class I to IV	Left ventricular EF<25%, NYHA class III and IV with symptoms of severe HF such as dyspnea, fatigue on a minimal amount of exertion, or at rest
Primary intervention tested	Bisoprolol 1.25 mg group (n=1327) vs. placebo group (n=1320) daily, the drug being used incrementally to a maximum dose of 10 mg per day.	Metoprolol CR/XL, with a dosage of 25 mg once a day for NYHA class II subjects, or 12.5 mg once per day for NYHA class III or IV subjects (n=1990), titrated for 6 to 8 weeks up to a target dosage of 200 mg once per day or placebo (n=2001).	Carvedilol (n=1156) or placebo (n=1133) with an initial dose of 3.125 mg taken twice every day for two weeks, which was then increased at progressive, two-week intervals unless otherwise required, first to 6.25 mg, then to 12.5 mg, and then to a target dose of 25 mg twice every day.
Study conclusion	In stable HF patients, BB therapy demonstrated survival benefits.	Metoprolol CR/XL had improved survival and reduced the hospitalization rates secondary to HF, showed improvement in NYHA functional class, and had a significant improvement in the quality of life of HF patients.	Carvedilol demonstrated morbidity and mortality benefits in patients with severe HF.

ACEIs and ARBs in heart failure

ACEIs and ARBs work by inhibiting the effects mediated by the RAAS pathway, through inhibiting the enzymatic activity of the angiotensin-converting enzyme (ACE) and preventing the formation of angiotensin II [26]. Heikkilä et al. conducted the Cooperative North Scandinavian Enalapril Survival Study (CONSENSUS trial) study in Northern Europe to determine whether enalapril (2.5 mg to 40 mg a day) had a beneficial effect on patients with severe HF [27]. The double-blind study, which enrolled 253 patients and had a mean follow-up of 188 days, concluded that enalapril reduced the crude mortality rate in the treatment group by 40% (p=0.002). The reduction in mortality rate was determined as 31% at one year. The study concluded that ACEIs reduced mortality and delayed the progression of HF.

This finding was further supported by the Studies of Left Ventricular Dysfunction (SOLVD) trial, a double-blind, randomized study conducted by Yusuf et al. [28]. The trial enrolled 2569 subjects, of which 1285 subjects were assigned to the enalapril group and 1284 subjects to the placebo group. The subjects were followed up for a mean duration of 41.4 months. The study concluded that enalapril significantly decreased HF incidence and hospitalizations related to HF compared to placebo.

Today, ACEIs are established, first-line drugs in HF and are proven to reduce mortality, delay cardiac remodeling, and improve quality of life in HF patients [1]. However, intolerance to ACEIs is common and that is where ARBS came into consideration [29].

ARBs work by physically blocking angiotensin receptors throughout the body [26]. Current guidelines recommend that ARBs be prescribed in the event of poor medication tolerance to ACEIs [1]. This is because ARBs have not demonstrated a consistently superior profile compared to ACEIs but remain an alternative to ACEI treatment [29]. The Valsartan Heart Failure Trial (Val-HeFT) conducted by Maggioni et al. concluded that in patients not treated with ACEIs, valsartan had an all-cause mortality and morbidity rate of 17.3% vs. 27.1% for placebo (p=0.017) [30]. Based on the outcome, valsartan, an ARB, appears as a practical and effective treatment choice in patients intolerant to ACEIs [30]. 

Because the Losartan Heart Failure Survival (ELITE II) study concluded that ACEI use resulted in significant side effects such as cough, Granger et al. conducted the randomized, double-blind, multicenter, Candesartan in Heart Failure Assessment of Reduction in Mortality and Morbidity (CHARM-Alternative) trial [31-32]. The study examined whether candesartan, an ARB, provided mortality benefit in patients intolerant to ACEIs. The trial enrolled 2028 subjects with an EF of 40% or less who were assigned to either candesartan or placebo. The subjects were followed up for a mean of 33.7 months. The results showed the candesartan group exhibited 33% cardiovascular deaths or hospitalizations vs. 40% in the placebo group, hazard ratio (unadjusted) 0.77 [95% CI 0.67-0.89]; p=0.0004. The study concluded that candesartan resulted in fewer side effects, showed greater tolerance than ACEIs, and reduced cardiovascular mortality and morbidity in patients with symptomatic HF.

A variant of this study was the Candesartan Cilexetil in Heart Failure Assessment of Reduction in Mortality and Morbidity (CHARM-Preserved) trial [33]. This study explored the benefit of adding ARB to the treatment regimen among patients with HFpEF. The study enrolled 3023 subjects with EF greater than 40%, of which 1514 were randomized to the candesartan group, and 1509 were randomized to matching placebo. The subjects were followed up for a mean of 36.6 months and concluded that among patients with HFpEF, candesartan, an ARB had a moderate role in preventing hospitalizations. 

These trials laid the foundation for the use of ACEIs and ARBs in HF and are the established first-line drugs in HF. The main side effects of ACEIs include angioedema and cough, whereas ARBs can result in hypotension, dysfunction of the renal system, and hyperkalemia [1]. Table 3 summarizes the studies discussed above.

**Table 3:** Trials that investigated the use of ACEIs and ARBs in heart failure CONSENSUS: "Effects of enalapril on mortality in severe congestive heart failure, results of the cooperative north Scandinavian enalapril survival study"; SOLVD: "Effect of enalapril on survival in patients with reduced left ventricular ejection fractions and congestive heart failure"; Val-HEFT: "A randomized trial of the angiotensin-receptor blocker valsartan in chronic heart failure"; CHARM alternative: "The effects of candesartan in patients with chronic heart failure and reduced left ventricular systolic function intolerant to angiotensin-converting-enzyme inhibitors: the CHARM-Alternative trial"; CHARM preserved: "The effect of candesartan on patients with chronic heart failure, and with preserved left ventricle ejection fraction: The CHARM-Preserved trial"; NYHA: "New York Heart Association"; HFrEF: Heart failure with reduced ejection fraction; ACEI: Angiotensin-converting enzyme inhibitor; EF: Ejection fraction; LVEF: Left ventricular ejection fraction; HFpEF: Heart failure with preserved ejection fraction; mg: milligram

Name of the Study	CONSENSUS, 1987 [27]	SOLVD, 1991 [28]	Val-HEFT, 2001 [30]	CHARM-Alternative, 2003 [32]	CHARM-Preserved, 2003 [33]
Type of study	Randomized, double-blind, controlled, parallel-group trial	Randomized, double-blind, placebo-controlled trial	Randomized, double-blind, controlled trial	Randomized, double-blind trial	Multicenter, randomized, double-blind and controlled, trial
Number of subjects	253	2569	5010	2028	3023
Mean follow-up duration	188 days	41.4 months	23 months	33.7 months	36.6 months
Patient profile	NYHA class IV, HFrEF	NYHA class II or III, not previously on an ACEI, EF ≤ 0.35	NYHA class II to IV	Symptomatic HF, LVEF <40%, not receiving ACEIs due to intolerance	NYHA class II to IV, LVEF >40%
Primary hypothesis tested	In patients with severe HF, does enalapril slow the prognosis and improve survival?	Does enalapril decrease mortality rates among patients with HFrEF when added to the therapy regimen?	Does valsartan provide mortality and morbidity benefits in patients with HFrEF when added to standard treatment regimen?	Does ARBs provide mortality benefit similar to ACEIs in patients unable to take ACEIs due to intolerance?	Does adding ARB to the treatment regimen of HFpEF provide mortality benefit compared to placebo?
Primary intervention tested	Enalapril (n=127) vs placebo (n=126)	Enalapril (n=1285) vs placebo (n=1284) at doses of 2.5 mg to 20 mg a day	Valsartan 160 mg daily (n= 2511) vs placebo twice daily (n= 2499)	Candesartan; targeted dose of 32 mg once daily (n=1013) or matching placebo (n=1015)	candesartan (n=1514), target dose of 32 mg once daily or placebo (n=1509).
Study conclusion	Enalapril use demonstrated a reduction in mortality and resulted in symptom improvement when added to standard therapy.	Adding enalapril to standard therapy reduced hospitalizations and mortality in patients with HFrEF.	When added to prescribed therapy, valsartan reduces mortality and morbidity and improves clinical signs and symptoms in patients with HF.	Candesartan exhibited good tolerance and reduced cardiovascular mortality and morbidity in symptomatic HF patients intolerant to ACEIs.	In subjects with HFpEF, candesartan had a moderate impact on reducing hospital admissions.

Angiotensin receptor neprilysin inhibitors (ARNI) and heart failure

In 2015, the US Food and Drug Administration (FDA) approved the combined angiotensin II and neprilysin inhibitor for HF patients [34]. Neprilysin is an inhibitor of natriuretic peptides such as atrial natriuretic peptide (ANP) [35]. Natriuretic peptides are a family of three primary compounds - ANP, brain natriuretic peptide (BNP), and C-type natriuretic peptide (CNP) [36]. The levels of these compounds rise in a pathologic state such as HF [37]. This increase is observed because the natriuretic peptides work to lower the heart's workload by promoting natriuresis and relaxation of the cardiac myocytes [35-36]. However, these compounds, particularly ANP, have a short half-life and are metabolized by endopeptidases [37]. Neprilysin inhibitors, such as sacubitril, halt the cleavage of ANP, increasing their half-life and promoting their effects [38]. However, neprilysin inhibitors alone did not demonstrate superiority compared to existing first-line drugs such as ACEIs [39].

The Angiotensin-Neprilysin Inhibition Versus Enalapril in Heart Failure (PARADIGM-HF) trial was the first study to compare ARNI and ACEIs and was conducted by Mc Murray et al. [40]. The study was designed as a double-blind, two-arm, randomized, parallel-group, event-driven trial enrolling 8442 subjects, conducted across 47 countries and 985 sites worldwide. The subjects enrolled patients with a left ventricular ejection fraction (LVEF) of <40% to <35% with a mean LVEF of 29% and NYHA classes I through IV. Subjects were randomized into LCZ696 (sacubitril or valsartan) group, taken 200 mg orally twice a day, and enalapril group, taken as 10 mg oral drug, twice a day. Mean follow-up accounted for 27 months, and the study ended earlier than anticipated because of significant beneficial findings. The study's primary outcomes-death or hospitalizations secondary to HF, for ARNI vs. ACI, was measured at 21.8% vs. 26.5%, the hazard ratio of 0.80 (95%CI 0.73-0.87, p<0.001). There were significant improvements in HF hospitalizations (12.8% vs 15.6%; p<0.001), all-cause mortality (17% vs 19.8%; p=0.0009), and cardiovascular-related death (13.3% vs 16.5%; p=0.001). The study concluded that among patients with HFrEF, sacubitril/valsartan showed a reduction in cardiovascular death and hospitalization secondary to HF. The intervention also slowed the worsening of HF and improved symptoms of HF.

As previously discussed, high levels of BNP correspond to pathological states such as HF. In 2019, Morrow et al. conducted the Comparison of Sacubitril/Valsartan Versus Enalapril on Effect on NT-proBNP in Patients Stabilized From an Acute Heart Failure Episode (PIONEER-HF) trial to study if sacubitril/valsartan reduced N-terminal Pro-b-type natriuretic peptide (NT-Pro-BNP) levels during acute HF decompensation [41]. The study enrolled 881 patients and was designed as a double-blind, randomized, multicenter, and active-controlled trial. Four-hundred forty subjects were assigned sacubitril/valsartan, with initial dosing of 26/49 mg twice a day taken orally, and 441 subjects assigned to enalapril group, with initial dosing of 2.5 mg, increased to 5 mg and 10 mg as a final target, taken twice a day. The study's primary outcome determined the geometric mean of proportional, time-averaged changes in NT-pro-BNP levels, compared from baseline through weeks four to eight, was 0.53 in the ARNI group and 0.75 in the ACEI group (46.7% vs. 25.3% percent change, p=<0.001). Beneficial secondary outcomes, such as improvement in HF-related rehospitalization, were also noted (8% vs. 13.8%; p<0.005). The study concluded that sacubitril/valsartan caused a more significant reduction in NT-Pro-BNP levels than enalapril alone among patients with acutely decompensated HF. However, further studies are required to elicit the clinical endpoints of decompensated HF and not laboratory endpoints.

Similarly, much research is needed to determine efficacious therapy in patients with HFpEF. Many studies, including the Prospective Comparison of ARNI with ARB Global Outcomes in HF with Preserved Ejection Fraction (PARAGON HF trial), conducted by Solomon et al., demonstrated that sacubitril/valsartan did not demonstrate a reduction in cardiovascular mortality or hospitalization rates in this patient profile [42]. Even though modest improvements were noted in NYHA status (15% vs. 12.6%; p=<0.05) for the sacubitril/valsartan vs. valsartan group, no significant improvements were noted in HF deaths or hospitalization rates (12.8 events in the sacubitril/valsartan group vs. 14.6 events in the valsartan group, p=not significant). The same study noted that ARNIs caused symptomatic hypotension as a side effect in many participants [42]. A summary of this study as well as the studies discussed above are listed in Table 4.

**Table 4:** Trials that investigated the use of ARNI in subjects with heart failure PARADIGM-HF: Angiotensin-neprilysin inhibition versus enalapril in heart failure; PIONEER-HF: Angiotensin-neprilysin inhibition in acute decompensated heart failure; PARAGON HF: Angiotensin–neprilysin inhibition in heart failure with preserved ejection fraction; NYHA: New York Heart Association; LVEF: Left ventricular ejection fraction; EF: Ejection fraction; ng: nanogram, ml: milliliter; NT-Pro-BNP: N-terminal prohormone of brain natriuretic peptide; ARNI: Angiotensin receptor-neprilysin inhibitor; HF: Heart failure; SCD: Sudden cardiac death; ACEI: Angiotensin-converting enzyme inhibitor; HFpEF: Heart failure with preserved ejection fraction; CI: Confidence interval

Name of the Study	PARADIGM-HF, 2014 [40]	PIONEER-HF, 2019 [41]	PARAGON-HF, 2019 [42]	
Type of study	Randomized, double-blind, event-driven, two-arm, parallel-group trial	Randomized, double-blind, active-controlled trial	Randomized, double-blind, active-comparator trial	
Number of subjects	8442	881	4822	
Mean/median Follow-up duration	27 months	8 weeks	35 months	
Patient profile	EF <40% to <35%, NYHA class I to IV	LVEF ≤40%, Pro BNP levels >1600 ng/ml, Hospitalized	EF ≥ 45%, NYHA class II to IV	
Primary outcome	Cardiovascular related death or hospitalization for heart failure occurred in 21.8% of the sacubitril/valsartan group vs. 26.5% of the enalapril group (p < 0.001)	Time-averaged decrease in the NT-proBNP concentration was significantly found to be greater in the sacubitril-valsartan (ARNI) group than in the enalapril (ACEI) group; the ratio of the geometric mean of values obtained at 4 and 8 weeks, to the baseline levels, was 0.53 in the sacubitril-valsartan group as compared with 0.75 in the enalapril group (percent change, -46.7% vs. -25.3%; the ratio of change with sacubitril-valsartan vs. enalapril, 0.71; 95% CI 0.63 to 0.81; P<0.001)	In the sacubitril/ valsartan group (n=526), 894 primary events were noted, accounting for 690 HF hospitalizations and 204 cardiovascular-related deaths. In the valsartan group (n=557), 1009 primary events were noted, accounting for 797 hospitalizations due to HF and cardiovascular-related death.	
Study conclusion	Sacubitril/Valsartan was associated with reducing cardiovascular death or hospitalizations secondary to HF, reduction in SCD, and slowed the progression of HF.	Sacubitril/valsartan led to a higher reduction in NT-proBNP levels compared to enalapril alone. However, the rates of side effects of the therapy, such as worsening renal function, hyperkalemia, symptomatic hypotension, and angioedema, did not significantly differ between the two groups.	Sacubitril/valsartan was not effective in lowering cardiovascular-related deaths and hospitalizations secondary to HF among HFpEF patients.	

Other treatment modalities for HF

Surgical approaches, such as heart transplants, remain one of the best options for tackling HF [43]. Although this article emphasized the importance of BBs, ACEI/ARBs, and ARNIs, drug classes, such as mineralocorticoid antagonists (MRAs) and sodium-glucose cotransporter 2 (SGLT-2) inhibitors, have also been shown to decrease hospitalizations and mortality in HF patients. Mineralocorticoid antagonists block aldosterone receptors, thereby preventing the deleterious effects of the RAA system. In 1999, Pitt et al. conducted a randomized, double-blind study, Effects of Spironolactone on the Morbidity and Mortality in Patients Suffering from Severe Heart Failure (RALES) trial to investigate if spironolactone prevented death and hospitalization in patients with severe HF and EF of less than or equal to 35% [44]. The study, which enrolled 1663 patients reported that at the end of the follow-up period (24 months), patients in the spironolactone group (n=822) reported 284 deaths (35%) compared to 386 deaths (46%) in the placebo group (n=841) (relative risk=0.70, p<0.001, 95% CI 0.60-0.82). The study also reported a 35% reduction in the frequency of hospitalizations in the spironolactone group and a significant level of symptomatic improvement, proving the efficacy of spironolactone in HFrEF. Additionally, the eplerenone in patients with systolic heart failure and mild symptoms study proved the efficacy of eplerenone in subjects with HFrEF and mild symptoms, thus establishing MRAs as routinely prescribed drugs in HF [45]. Furthermore, Pitt et al. investigated whether MRAs such as spironolactone efficiently treated HF with preserved ejection fraction [46]. They conducted a randomized, double-blind study, namely, the Spironolactone for Heart Failure with Preserved Ejection Fraction (TOPCAT trial), and reported that spironolactone reduced the incidence of hospitalizations by 12% compared to 14.2% in the placebo group (hazard ratio 0.83; 95% CI 0.69-0.99; P=0.04). However, the study concluded that in patients with HFpEF, spironolactone did not cause a significant reduction in cardiovascular deaths. MRAs are now proven to be efficacious and add significant clinical value in the treatment of HF. Adverse effects of MRAs include electrolyte imbalance, particularly hyperkalemia [1].

The sodium-glucose cotransporter 2 (SGLT-2) inhibitors were first marketed as anti-diabetic drugs but also have cardio-protective effects. In patients with HF and type 2 DM, the Empagliflozin, Cardiovascular Outcomes, and Mortality in Type 2 Diabetes (EMPA-Reg) trial showed that empagliflozin, an SGLT-2 inhibitor decreased cardiovascular-related mortality compared to placebo (10.5% vs. 12.1%; hazard ratio 0.86, 95%CI 0.74-0.99 and p=0.04) [47]. Furthermore, the Canagliflozin and Cardiovascular and Renal Events in Type 2 Diabetes (CANVAS) trial demonstrated that SGLT-2 inhibitors reduced HF hospitalizations and decreased cardiovascular deaths in patients with comorbid type 2 DM [48]. Because type 2 DM patients are also highly prone to developing HFpEF, SGLT-2 inhibitors have gained particular prominence in this subset of patients as an effective means of tackling HF-related complications [49]. The notable side effects of SGLT-2 inhibitors include increased risk of urinary tract infections (UTIs) and hypoglycemia [47].

Device therapy

Additionally, nonpharmacologic device therapies, such as implantable cardioverter defibrillators (ICDs), and cardiac resynchronization therapy (CRT) have also been approved by the American College of Cardiology (ACC) in the treatment of HF [1]. Patients with HF are at a significantly high risk of developing arrhythmia because of structural changes to the myocardium as a result of HF progression [1]. Hence, these devices significantly reduce the incidence of arrhythmia-induced sudden cardiac death (SCD) and decrease mortality in HF patients (Table 5) [50]. The ACC has made significant strides to establish recommendations (designated by classes I, II, and III) on the eligibility of the device therapy [1]. Table 5 summarizes some of the highlights of ACC recommendations on the role of ICD and CRT in HF treatment.

**Table 5:** Recommendations for ICD and CRT use in patients with HF ICD: Implantable cardioverter-defibrillator, CRT: Cardiac resynchronization therapy, SCD: Sudden cardiac death, LVEF: Left ventricular ejection fraction, NYHA: New York Heart Association, MI: Myocardial infarction, LBBB: Left bundle branch block, ms: milliseconds

Criteria	Implantable Cardioverter-Defibrillator (ICD) [1]	Cardiac Resynchronization Therapy (CRT) [1]
Recommended use (Class I)	Primary prevention of SCD in patients with LVEF ≤35%, NYHA class II/III symptoms; on standard medical therapy, at least more than 40 days after recent MI and >1 year survival expectancy	Patients with LVEF ≤35%, QRS duration of 150ms or more, on standard medical therapy, with sinus rhythm or LBBB morphology
Benefit unknown and not recommended (class II and class III)	ICD use provides uncertain benefit in HF patients with other non-cardiac comorbidities such as malignancy and patients with a high risk of non-sudden cardiac death	CRT is not recommended for patients with QRS ≤150ms; patients with non-LBBB morphology that belong to NYHA class I or II.

Symptomatic treatment

Symptomatic treatment is crucial for tackling HF since HF is a chronic condition. HF symptoms can be highly debilitating and can negatively impact the quality of life [1]. Non-pharmacologic symptom control strategies, such as patient education, reduced-sodium consumption (<2 grams/day), and low to moderate impact exercise, provide relief of symptoms from fluid overload and help patients have better control over their health [1,51]. Furthermore, several drugs not mentioned above can also aid in controlling the symptoms of HF and improve quality of life (Table 6) [1,51]. Table 6 lists the drugs, their mechanism of action, adverse effects, and clinical implications in the symptomatic treatment of HF [52-55].

**Table 6:** Pharmacologic properties and clinical implications of HF drugs HF: Heart failure, SOB: Shortness of breath, ATPase: Adenosine triphosphatase, GI: Gastrointestinal, If: Funny sodium channels, NYHA: New York Heart Association

Name of the drug	Mechanism of action	Clinical implication	Adverse reactions and Long-term side effects
Loop diuretics	Inhibit reabsorption of Sodium (Na) and Chloride (Cl) in the nephrons	Most effective drugs at improving HF symptoms such as peripheral fluid retention, SOB and also improve exercise tolerance	Hypotension, electrolyte disturbances such as hypomagnesemia, hypocalcemia, hypotension
Digoxin	Positive inotropic agent; inhibits Sodium-Potassium ATPase pump	Improves SOB and decreases the frequency of HF-related hospitalizations	Neuro-visual disturbance, GI disturbance, digoxin toxicity
Ivabradine	Inhibits funny sodium channels in the sino-atrial node (I-f channels)	Reduces heart rate and decreases HF-related hospitalizations	Neuro-visual disturbances, bradycardia
Hydralazine with Nitrates	Increases relaxation of arterial smooth muscle, thereby decreasing afterload	Improves HF symptoms and decreases mortality, particularly among African American patients falling in NYHA classes III and IV.	Headaches, nausea, fatigue, hypotension

Limitations

Although neurohormonal activation is the principal driver of pathophysiology seen in HF, several other intricate mechanisms are involved in the disease progression of HF, which this article could not fully delve into. This article did not mention all the trials that were pivotal in the field of HF, and trials involving diastolic HF could not be fully explored.

## Conclusions

Heart failure is a chronic syndrome that heterogeneously affects the human body. The condition manifests in several ways depending on the cause, but common symptoms include fatigue, shortness of breath, palpitations, chest pain, etc. As evident from the review, patients with heart failure benefit from first-line drugs such as BBs, ACEIs/ARBs, and the newer ARNIs. This article has summarized some of the most prominent randomized controlled trials in the realm of heart failure and concisely presented them. We believe that this review conveys the objectives, data, and results of these trials in a digestible manner and translates this information as to how it is clinically pertinent. The clinical implications of this article revolve around understanding the data behind the landmark trials in the field of heart failure. We believe that understanding this material can allow medical professionals to communicate effectively with patients. Countless experiments and multicenter trials have demonstrated the efficacy of the first-line drugs in treating heart failure and improving the quality of life in this patient population, particularly those with reduced ejection fraction. However, approximately half of patients with heart failure are diagnosed with heart failure with preserved ejection fraction, but little is known about the efficacy of first-line drugs in this group, which calls for an urgent need for further studies in this area. Finally, there is a dire need to define, classify, and develop a universal, data-driven algorithm to treat heart failure for the best possible patient outcomes.
